# Association of Glutathione S-transferase Gene Polymorphisms With Hepatocellular Carcinoma in Patients From Maharashtra, India

**DOI:** 10.7759/cureus.109473

**Published:** 2026-05-23

**Authors:** Shivani R Kale, Geeta Karande, Pratik P Durgawale, Aishwarya Garud, Anand Gudur, Rashmi Gudur, Satish Patil

**Affiliations:** 1 Molecular Biology and Genetics, Krishna Institute of Medical Sciences, Krishna Vishwa Vidyapeeth (Deemed to be University), Karad, IND; 2 Department of Microbiology, Krishna Institute of Medical Sciences, Krishna Vishwa Vidyapeeth (Deemed to be University), Karad, IND; 3 Department of Oncology, Krishna Institute of Medical Sciences, Krishna Vishwa Vidyapeeth (Deemed to be University), Karad, IND; 4 Oncology, Krishna Charitable Hospital, Karad, IND

**Keywords:** genetic polymorphism, gst, hepatocellular carcinoma, indian population, susceptibility

## Abstract

Background: Hepatocellular carcinoma (HCC) is a major global health burden with high mortality. Genetic variations in detoxification enzymes, particularly glutathione S-transferases (GSTs), may influence predisposition to HCC. This population-specific study investigates the relationship between GST gene polymorphisms and the occurrence of HCC in India.

Methods: A case-control study was conducted involving 380 individuals (190 confirmed HCC patients and 190 age- and gender-matched healthy controls) from India. Genotyping for GSTM1 (625 bp and 215 bp) and GSTT1 variants was performed using polymerase chain reaction (PCR), while the GSTP1 Ile105Val (A→G) polymorphism was analyzed using polymerase chain reaction-restriction fragment length polymorphism (PCR-RFLP). Associations between genotypes and the occurrence of HCC were evaluated using the chi-square test and odds ratio (OR) with a 95% confidence interval (CI); a p-value of ≤0.05 was considered statistically significant.

Results: The GSTT1 (480 bp) null genotype exhibited a statistically significant positive association with the occurrence of HCC (OR=2.681, 95% CI=1.73-4.14; p<0.0001). In contrast, the GSTM1 (215 bp) null genotype demonstrated a significant negative association with the occurrence of HCC (OR=0.135, 95% CI=0.072-0.25; p<0.0001). Similarly, the GSTM1 (625 bp) null genotype also showed a significant negative association with the occurrence of HCC (OR=0.5476, 95% CI=0.364-0.825; p=0.004). GSTP1 heterozygous (adjusted OR=0.517; 95% CI: 0.124-2.15; p=0.364) and variant (adjusted OR=1.28; 95% CI: 0.3-5.49; p=0.737) genotypes showed no significant association with HCC risk after adjustment for confounding factors.

Conclusion: The findings indicate that the GSTT1 (480 bp) null genotype is significantly associated with increased susceptibility to HCC in the Maharashtrian population. In contrast, GSTM1 null genotypes (215 bp and 625 bp) appear to confer a protective effect. GSTP1 polymorphism was not significantly associated with HCC risk after adjustment.

## Introduction

Hepatocellular carcinoma (HCC) is a growing global public health challenge, with incidence projected to rise steadily in the coming decades [1]. In India, it represents a leading cause of cancer-related mortality and healthcare expenditure, particularly among those with chronic liver disease [2]. Liver cancer accounted for 37,796 deaths in 2024, with a crude mortality rate of 2.7 per 100,000 population [3], figures that likely underestimate the true burden because of limited registry coverage in rural areas [4]. The high incidence-to-mortality ratio reflects its aggressive course and poor prognosis. In India, HCC has an estimated incidence, prevalence, and mortality rate of 2.15, 2.27, and 2.21 per 100,000 people, respectively. Furthermore, the projected number of incident liver cancer cases in India is expected to reach approximately 44,303 by 2025, highlighting the growing public health burden of the disease. The most commonly associated risk factors are aflatoxin exposure, nonalcoholic fatty liver disease (NAFLD), metabolic dysfunction-associated fatty liver disease (MAFLD), and infection with either the hepatitis B virus (HBV) or the hepatitis C virus (HCV) [5,6]. Despite this rising burden, epidemiological data specific to Maharashtra remain sparse. This regional gap in evidence underscores the need for dedicated investigations into the molecular epidemiological profile of HCC in this population, forming the primary motivation for the present study.

As the body’s principal site of biotransformation, the liver processes both internally generated metabolites and externally derived xenobiotics, rendering it uniquely vulnerable to accumulated damage from environmental pollutants, reactive metabolic intermediates, and chemical carcinogens [7]. The efficiency of these detoxification pathways plays a vital role in preventing malignant transformation. Several enzyme families, including glutathione S-transferases (GSTs), phase I cytochrome P450 enzymes (CYP1A, CYP2C, and CYP3A subfamilies), N-acetyltransferases (NATs), and UDP-glucuronosyltransferases (UGTs), are central to neutralizing and eliminating harmful compounds [8]. Genetic polymorphisms within these detoxification genes can reduce or alter enzyme activity, potentially impairing the metabolism of carcinogens and thereby increasing susceptibility to hepatocarcinogenesis [9,10]. Among these families, GST enzymes are particularly important in the liver’s phase II detoxification system. The GST superfamily includes GSTM1 (mu), GSTT1 (theta), and GSTP1 (pi), which catalyze the conjugation of glutathione with a broad spectrum of toxic electrophiles, including drugs, environmental contaminants, carcinogens, and reactive oxygen species (ROS) [11]. GSTM1 is the predominant GST isoform in the liver and is responsible for detoxifying aflatoxin B1 and other hepatotoxic carcinogens; GSTT1 metabolizes small epoxides and halogenated compounds commonly encountered in environmental and occupational exposures; and GSTP1 modulates oxidative stress responses and is highly expressed in hepatic tissue [11,12]. Homozygous deletion in GSTM1 and GSTT1, referred to as “null genotypes,” may result in complete loss of enzymatic activity, potentially weakening the liver’s protective capacity [12,13].

Numerous population-based investigations have evaluated how GST gene variants relate to HCC risk, with particular emphasis on Asian cohorts [14]. Meta-analyses further support these findings, indicating significantly elevated odds of HCC among carriers of these null variants, with the strongest associations observed in Asian and Indian populations [15-17]. Beyond gene deletions, SNPs (single nucleotide polymorphisms) in genes like GSTP1 can alter enzyme expression and activity, contributing to individual variation in detoxification capacity [18,19]. Furthermore, studies investigating the association between genetic variations and the occurrence of HCC are particularly rare within the Indian population. Such knowledge is crucial for identifying individuals at elevated risk and could contribute to improved screening and preventive strategies. Therefore, the primary objective of this case-control study was to investigate the association between GSTM1, GSTT1, and GSTP1 gene polymorphisms and HCC susceptibility in the Maharashtrian population. This study hypothesized that GSTM1, GSTT1, and GSTP1 gene polymorphisms are associated with susceptibility to HCC in the Maharashtrian population.

## Materials and methods

In the present case-control study, 190 histopathologically confirmed HCC cases were recruited from the Department of Oncology, Krishna Hospital and Medical Research Centre, Karad, between 2023 and 2025. Patients with any other primary cancer and individuals unwilling to provide written informed consent were excluded from the study.

The control group comprised healthy, cancer-free, sex-matched individuals recruited from the same region. Individuals with a previous or current history of cancer and those unwilling to provide written informed consent were excluded from the control group.

Demographic and clinical data were collected from all participants using a structured questionnaire, which included information on age, sex, diet, habits, and family history of cancer. Written informed consent was obtained from all participants before enrollment, following a thorough explanation of the study objectives, procedures, and their right to withdraw at any time without consequence. The study protocol was reviewed and approved by the Institutional Ethics Committee of Krishna Institute of Medical Sciences (IEC No. 461/2022-2023).

Genomic DNA isolation from whole blood

A volume of 2-3 mL of peripheral blood was collected from both HCC cases and healthy controls using sterile ethylenediaminetetraacetic acid (EDTA)-coated vacutainers. DNA was extracted from all collected samples using the DNA Extraction Kit (Blood DNA Mini Kit, Make: Omega Biotek E.Z.N.A.) following the manufacturer’s protocol.

Genotyping assays

**Figure 1:**
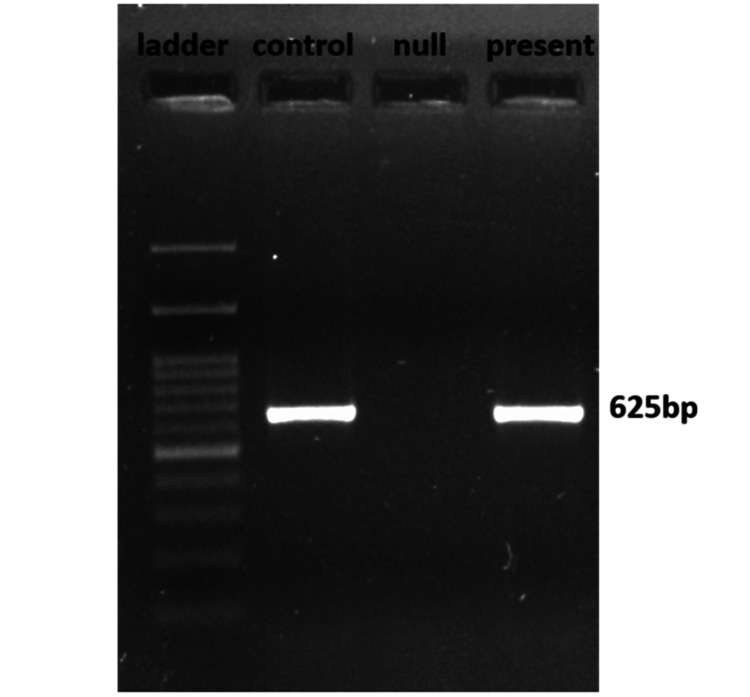
GSTM1 (625 bp) polymorphism Lane 1=100 bp DNA ladder; Lane 2=positive control; Lane 3=null genotype; Lane 4=present genotype (625 bp band)

**Figure 2:**
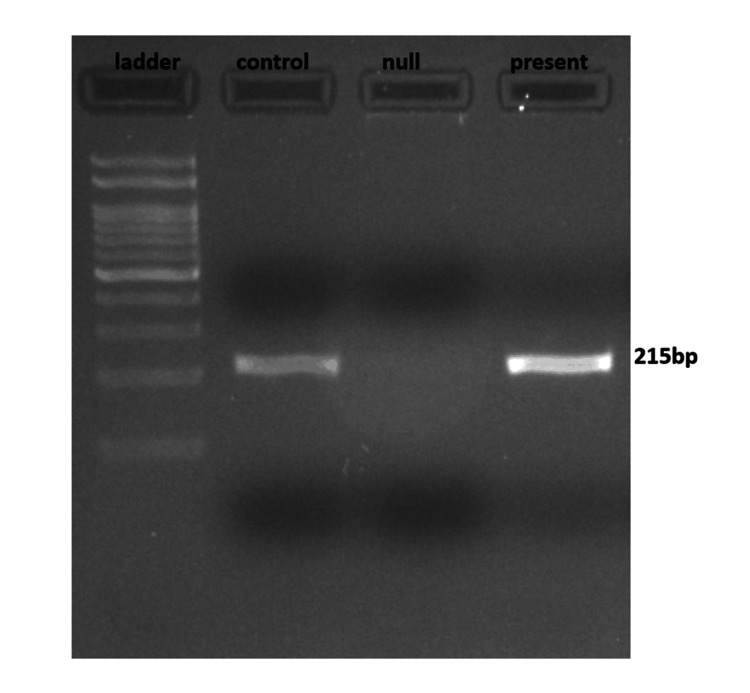
GSTM1 (215 bp) polymorphism Lane 1=100 bp DNA ladder; Lane 2=positive control; Lane 3=null genotype; Lane 4=present genotype (215 bp band)

**Figure 3:**
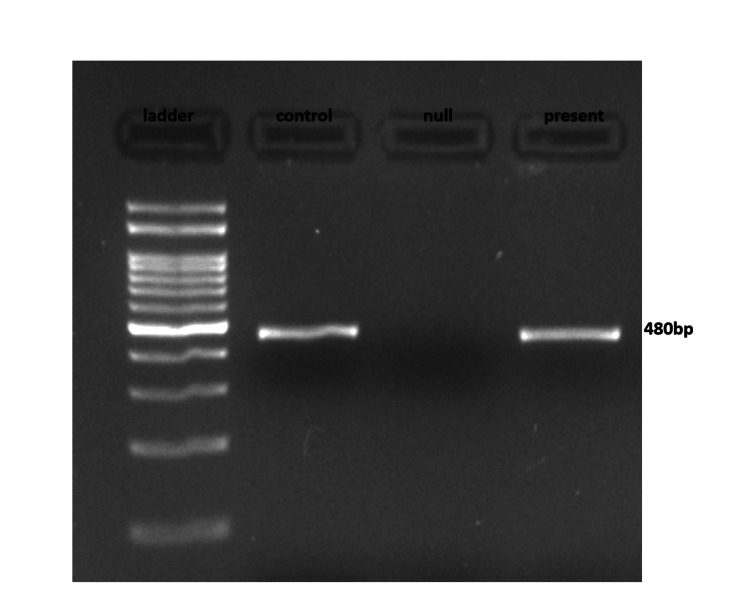
GSTT1 (480 bp) polymorphism Lane 1=100 bp DNA ladder; Lane 2=positive control; Lane 3=null genotype; Lane 4=present genotype (480 bp band)

Genotyping of GST genes was carried out using polymerase chain reaction (PCR), followed by restriction fragment length polymorphism (RFLP) analysis. The GSTM1 (215 bp) and GSTT1 genes were amplified using PCR. PCR amplifications were carried out in a total reaction volume of 20μL containing genomic DNA, primers, deoxynucleotide triphosphates (dNTPs), magnesium chloride (MgCl₂), buffer, and Taq DNA polymerase. The primer sequences, restriction enzymes, and digested band sizes are listed in Table 1 [10]. For GSTM1, a 625 bp (Figure 1) fragment was amplified using the following thermal cycling conditions: initial denaturation at 95°C for 5 minutes, followed by 30 cycles of 95°C for 30 seconds, 56°C for 30 seconds, and 72°C for 30 seconds, with a final extension step at 72°C for 5 minutes. For GSTM1, a 215 bp (Figure 2) PCR amplification was performed using the same program with an annealing temperature of 54°C. Amplification of the GSTT1 gene, 480 bp (Figure 3), was carried out under similar conditions, with annealing at 60°C for 30 seconds. A homozygous null genotype (nonfunctional allele) for GSTM1 and GSTT1 was inferred by the absence of a PCR product, whereas the presence of an amplified fragment indicated the gene’s presence. Previously validated DNA samples with known GSTM1 and GSTT1 genotypes were included as internal laboratory controls in each PCR run. To ensure genotyping reliability, approximately 10% of samples were randomly regenotyped independently, yielding 100% concordance with the original results.

**Table 1:** List of GST genes with detailed RFLP procedures, including primers, restriction enzymes, and expected products of selected genes PCR, polymerase chain reaction; RFLP, restriction fragment length polymorphism; GST, glutathione S-transferase; bp, base pair; FP, forward primer; RP, reverse primer; ref, reference; hr, hour; U, unit; NA, not applicable

Gene	rs no.	Amino acid/nucleotide change	Primer sequence forward/reverse	PCR product	Enzyme and digestion conditions	Wild type	Heterozygous	Mutant	
GSTP1 exon-5 codon-105 (A313G)	rs1695	Ile105Val (A>G)	FP: 5’-GTA GTT TGC CCA AGG TCA AG-3’ RP: 5’-AGC CAC CTG AGG GGT AAG -3’	433 bp	1 U of BsmA1 at 37°C for 2 hr	AA: 328bp, 105bp	A/G: 328 bp 222 bp 105 bp 106 bp	G/G: 222 bp, 105 bp 106 bp	
GSTM1		GSTM1 Null	FP: 5'–CAA ATT CTG GAT TGT AGC AGA TCA TGC–3' RP: 5'–CAC AGC TCC TGA TTA TGA CAG AAG CC–3'	625 bp	NIL	625 bp Present	NA	No amplification, null genotype	
GSTM1		GSTM1 Null	FP: 5’-GAA CTC CCT GAA AAG CTA AAG C-3’ RP: 5'-GTT GGG CTC AAA TAT ACG GTG G-3’	215bp	NIL	215 bp Present	NA	No amplification, null genotype	
GSTT1		GSTT1 Null	FP: 5’-TTC CTT ACT GGT CCT CAC ATC TC-3’ RP: 5’-TCA CCG GAT CAT GGC CAG CA-3’	480bp	NIL	480 bp Present	NA	No amplification, null genotype	

**Figure 4:**
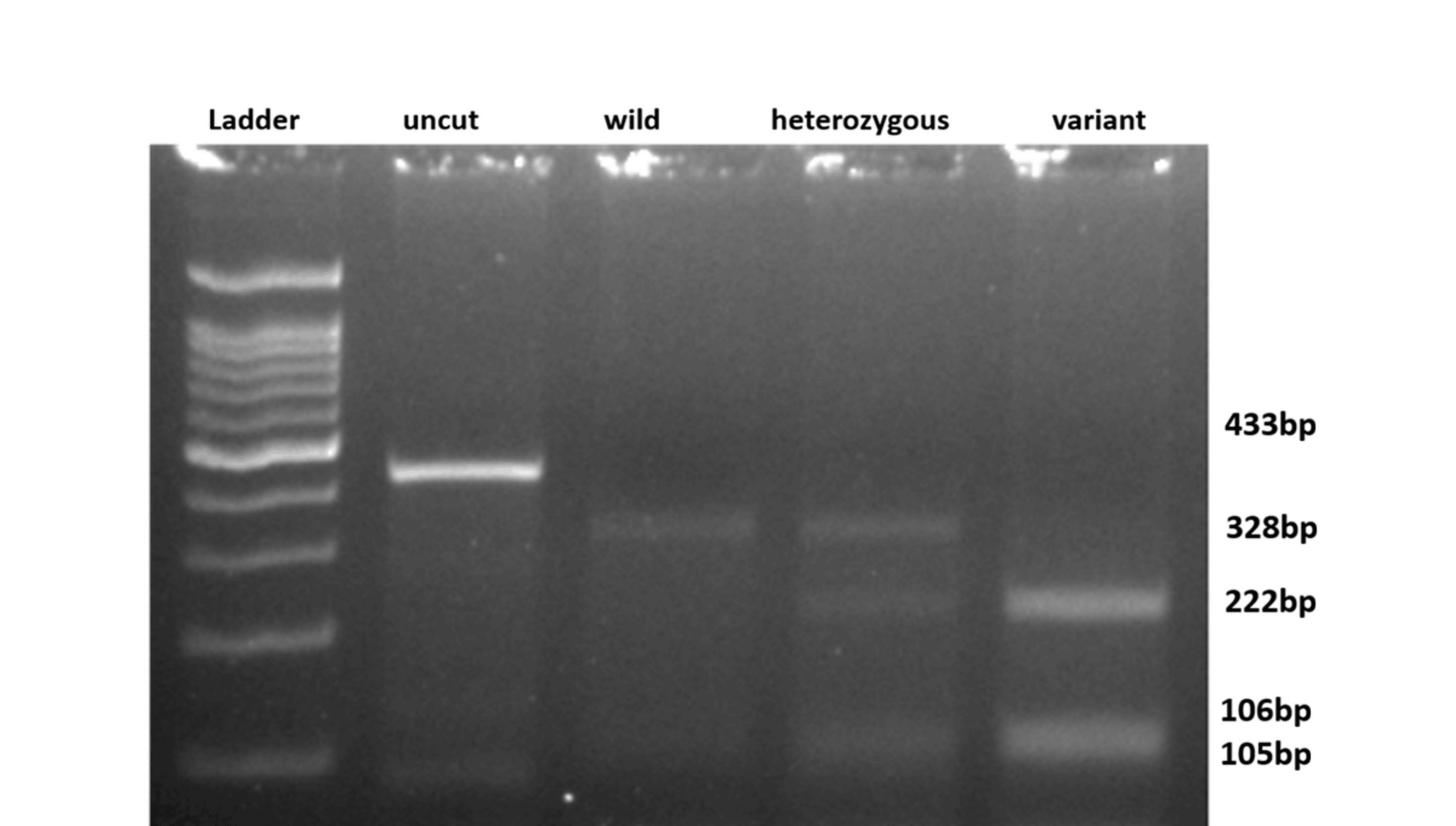
GSTP1 (433 bp) polymorphism Lane 1=100 bp DNA ladder; Lane 2=undigested PCR product (433 bp); Lane 3=homozygous wild-type genotype (A/A: 328 bp and 105 bp fragments); Lane 4=heterozygous genotype (A/G: 328 bp, 222 bp, 105 bp, and 106 bp fragments); Lane 5=homozygous variant genotype (G/G: 222 bp, 105 bp, and 106 bp fragments). PCR, polymerase chain reaction

Genotyping of GSTP1 Ile105Val, exon 5 (rs1695), was achieved using PCR followed by restriction fragment length polymorphism (PCR-RFLP) analysis using specific primers, as indicated in Table 1. The PCR conditions for amplifying the 433 bp fragment of GSTP1 (Figure 4) included an initial denaturation at 95°C for 5 minutes, followed by 30 cycles of 95°C for 20 seconds, 55°C for 20 seconds, and 72°C for 20 seconds, ending with a final extension at 72°C for 10 minutes. The resulting PCR products were then subjected to 1 U of BsmA1 restriction enzyme at 37°C for 2 hours. Digested and undigested PCR products were resolved by agarose gel electrophoresis on a 2% agarose gel at 100 V for 45 minutes using Tris-acetate-EDTA (TAE) buffer. Gels were stained with ethidium bromide (10 mg/mL), visualized under a UV transilluminator, and documented using a gel documentation system.

Statistical analysis

Allele and genotype frequencies for the GSTP1 polymorphism were calculated and tested for their distribution according to the Hardy-Weinberg equilibrium. The association between the GSTM1 (625 bp and 215 bp), GSTT1, and GSTP1 genotypes and the occurrence of HCC was studied using a binary logistic regression model with adjustment for confounding variables using SPSS version 27 (SPSS Inc., Chicago, IL, USA). Demographic and lifestyle factors that showed significant differences in distribution between cases and controls (age, family history of cancer, and habits) were included as confounding variables in the adjusted logistic regression analysis. The odds ratio (OR) and 95% confidence intervals (CI) were calculated to determine the occurrence of HCC associated with genotypes. A p-value of ≤0.05 was considered statistically significant.

Sociodemographic and lifestyle characteristics of cases and controls were compared using the chi-square (χ²) test for categorical variables such as age, gender, dietary habits, family history of cancer, and tobacco/alcohol use. Fisher’s exact test was used to assess the association of the GSTP1 variant genotype (G/G) with HCC occurrence, as the expected count in that cell was less than five. Potential confounders were identified, including age group, diet, habits, and family history of cancer. Binary logistic regression with simultaneous entry of these confounders was performed to calculate adjusted ORs and 95% CI for each GST genotype. A p-value of ≤0.05 was considered statistically significant for all analyses.

## Results

Sociodemographic and lifestyle characteristics of the study population

Sociodemographic characteristics of the 190 HCC patients and 190 controls are presented in Table 2. The age distribution in cases and controls differed significantly (p=0.005), whereas the gender distribution did not (p=0.817). A positive family history of cancer was significantly higher among cases compared to controls (p=0.0001). Regarding lifestyle factors, tobacco use and combined alcohol and tobacco consumption were significantly more prevalent among HCC cases (p=0.0001), indicating a strong association with disease occurrence.

**Table 2:** Sociodemographic characteristics, lifestyle factors, and GSTM1, GSTT1, and GSTP1 genotype distribution among HCC cases and controls Significance p<0.05 HCC, hepatocellular carcinoma

Variables		Cases (n=190)	Controls (n=190)	Chi-squared value	p-value
Age	18-40 years	20 (10.5%)	42 (22.1%)	10.687	0.005
	41-60 years	75 (39.5%)	75 (39.5%)		
	Above 61 years	95 (50%)	73 (38.4%)		
Gender	Male	138 (72.6%)	140 (73.7%)	0.054	0.817
	Female	52 (27.4%)	50 (26.3%)		
Diet	Vegetarian	10 (5.3%)	20 (10.5%)	3.62	0.057
	Mixed	180 (94.7%)	170 (89.5%)		
Family history of cancer	Yes	38 (20%)	10 (5.3%)	18.695	0.0001
	No	152 (80%)	180 (94.7%)		
Habits	Tobacco	79 (41.6%)	34 (17.9%)	173.257	0.0001
	Alcohol	8 (4.2%)	0 (0%)		
	Both	69 (36.3%)	0 (0%)		
	None	34 (17.9%)	156 (82.1%)		

After adjustment, family history of cancer (adjusted OR=5.047; 95% CI: 1.850-13.772; p=0.002) was identified as a significant confounding factor, whereas age group, diet, and habits did not show significant associations with HCC occurrence in the adjusted model.

Analysis of GSTM1 (625 bp), GSTM1 (215 bp), GSTT1, and GSTP1 (A313G) polymorphisms in GST genes

The distribution of genetic frequencies of GSTM1, GSTT1, and GSTP1 genotypes in HCC cases and controls (age- and gender-matched) was analyzed using logistic regression analysis.

The distribution of GSTM1 (625 bp) genotypes differed significantly between HCC cases and controls (χ²=8.365, p=0.004). The null genotype was observed less frequently in cases (36.8%) than in controls (51.6%), demonstrating a significant negative association with HCC occurrence (OR=0.5476; 95% CI: 0.364-0.825). The GSTM1 (215 bp) null genotype was also significantly less frequent in HCC cases (7.4%) compared to controls (37%) (χ²=48.345, p<0.0001), demonstrating reduced odds of HCC occurrence (OR=0.135; 95% CI: 0.072-0.25) (Table 3). The GSTT1 null genotype was significantly more frequent in HCC cases (46.8%) compared to controls (24.47%) (χ²=20.2, p<0.0001), demonstrating a strong positive association with HCC occurrence (OR=2.681; 95% CI: 1.73-4.145) (Table 3).

**Table 3:** Analysis of genotype frequency distribution of GSTM1 (625 bp), GSTM1 (215 bp), GSTT1, and GSTP1 genes in HCC cases and controls *Fisher’s exact test CI, confidence interval; 1, reference; OR, odds ratio; HCC, hepatocellular carcinoma

Gene	Genotype	Cases (N=190)	Controls (N=190)	Chi-square	OR (95% Cl)	P-value	Adjusted OR (95% Cl)	P-value
GSTM1 625 bp	Presence	120 (63.2%)	92 (48.4%)	8.365	1	0.004	1	0.005
	Null	70 (36.8%)	98 (51.6%)		0.5476 (0.364-0.825)		0.387 (0.2-0.745)	
GSTM1 215 bp	Presence	176 (92.6%)	119 (63%)	48.345	1	0.0001	1	0.003
	Null	14 (7.4%)	70 (37%)		0.135 (0.0728-0.25)		0.251 (0.102-0.621)	
GSTT1 (480 bp)	Presence	101 (53.2%)	143 (75.3%)	20.2	1	0.0001	1	<0.001
	Null	89 (46.8%)	47 (24.7%)		2.681 (1.73-4.145)		4.81 (2.43-9.52)	
GSTP1 (433 bp)	Wild type	132 (69.5%)	106 (55.8%)	8.820 (p=0.012)	1		1	
	Heterozygous	48 (25.3%)	75 (39.5%)		0.514 (0.329-0.801)	0.0038	0.517 (0.124-2.15)	0.364
	Variant type	10 (5.3%)	9 (4.7%)		0.892 (0.349-2.276)	0.8155*	1.28 (0.3-5.49)	0.737

GSTP1 genotype distribution differed significantly between cases and controls (χ²=8.820, p=0.012). On crude analysis, the heterozygous genotype showed a significant negative association with HCC occurrence (OR=0.514; 95% CI: 0.329-0.801; p=0.0038); however, this association was lost after adjustment for confounders (adjusted OR=0.517; 95% CI: 0.124-2.15; p=0.364). Similarly, the variant genotype showed no significant association in either crude (OR=0.892; 95% CI: 0.349-2.276; p=0.8155) or adjusted analysis (adjusted OR=1.28; 95% CI: 0.3-5.49; p=0.737) (Table 3).

## Discussion

Genetic susceptibility to HCC is highly heterogeneous, involving multiple rare and largely unlinked genetic alterations that may elevate disease susceptibility. Despite growing research, the overall contribution of this genetic architecture to individual susceptibility remains incompletely understood. GSTs constitute a major class of phase II detoxification enzymes that play a crucial role in the metabolism and elimination of carcinogens, environmental pollutants, and therapeutic drugs. Polymorphisms in GST genes, particularly GSTM1, GSTT1, and GSTP1, are associated with absent or reduced enzymatic activity, thereby compromising detoxification capacity and increasing susceptibility to various cancers, including bladder [16], oral [17], prostate [18], gastric [19], and cervical [20] cancers. However, the role of these polymorphisms in HCC remains relatively underexplored, with limited and inconsistent evidence available in the literature. To the best of our knowledge, this is the first study to investigate the association of GST gene polymorphisms (GSTM1, GSTT1, and GSTP1) with HCC susceptibility in the population of Maharashtra, India.

The GSTM1 null genotype has been widely reported to be associated with increased HCC susceptibility in several populations [21,22]. However, in the present study, a negative association was observed. Since this finding differs from many previous studies, it should be interpreted with caution and requires further validation in larger populations. In contrast, the GSTT1 null genotype may be associated with HCC susceptibility in the present study. This finding is consistent with results reported from Tamil Nadu [21] and Northeast India [22]. The convergence of these findings across geographically and ethnically distinct Indian populations strengthens the evidence that the GSTT1 null genotype may be associated with increased HCC susceptibility in the Indian subcontinent. However, further large-scale and mechanistic studies are needed to validate these findings and clarify their potential clinical relevance. Regarding GSTP1 polymorphism, no statistically significant association with HCC susceptibility was observed after adjusting for confounding factors. These findings are consistent with studies conducted in China [23,24] and Japan [25]. However, the low frequency of GSTP1 variant genotypes in the present study may have limited the statistical power to detect significant associations. Therefore, the findings should be interpreted cautiously, and larger studies are needed to further clarify the role of GSTP1 polymorphism in HCC susceptibility. Several studies conducted in different regions have investigated the relationship between GSTM1, GSTT1, and GSTP1 polymorphisms and HCC; however, their results have been inconsistent. These differences may be due to variations in sample size, dietary patterns, environmental exposures, and genetic diversity among populations. This is a single-center, area-specific study; hence, it cannot be generalized to the Indian population. Larger studies are needed to confirm these results and to better understand the role of gene-environment interactions. Additionally, well-designed studies across different geographical regions of India are essential to clearly define the contribution of GST polymorphisms to HCC occurrence and determine their clinical significance in cancer susceptibility.

Although this study provides valuable insights, certain limitations should be acknowledged, including potential recall bias due to self-reported lifestyle and environmental exposure data and possible selection bias. Future studies with larger cohorts and integrated genetic and clinical risk models with functional assays are needed to validate and strengthen these findings regarding HCC susceptibility.

## Conclusions

In conclusion, the present study demonstrates a significant association between the GSTT1 (480 bp) null genotype and increased susceptibility to HCC. Conversely, GSTM1 null genotypes (625 bp and 215 bp) may be negatively associated with the occurrence of HCC. However, this observation is inconsistent with previously reported findings, and validation across diverse study populations is warranted before definitive conclusions can be drawn. Furthermore, GSTP1 polymorphism did not show an association with HCC risk after adjustment, suggesting a limited role in HCC susceptibility in this population.

Given the multifactorial nature of HCC, individual polymorphisms may have limited predictive and standalone clinical utility. Therefore, larger-scale studies incorporating polygenic, environmental, and clinical risk factors are needed to validate and strengthen these associations.
